# Identification and Expression Analysis of *Medicago truncatula Isopentenyl Transferase Genes* (*IPT*s) Involved in Local and Systemic Control of Nodulation

**DOI:** 10.3389/fpls.2018.00304

**Published:** 2018-03-09

**Authors:** Mahboobeh Azarakhsh, Maria A. Lebedeva, Lyudmila A. Lutova

**Affiliations:** Department of Genetics and Biotechnology, Saint Petersburg State University, Saint Petersburg, Russia

**Keywords:** cytokinin, IPT, legume-rhizobium symbiosis, nodule development, autoregulation of nodulation

## Abstract

Cytokinins are essential for legume plants to establish a nitrogen-fixing symbiosis with rhizobia. Recently, the expression level of cytokinin biosynthesis *IPT*s (*ISOPENTENYLTRANSFERASES*) genes was shown to be increased in response to rhizobial inoculation in *Lotus japonicus, Medicago truncatula* and *Pisum sativum*. In addition to its well-established positive role in nodule primordium initiation in root cortex, cytokinin negatively regulates infection processes in the epidermis. Moreover, it was reported that shoot-derived cytokinin inhibits the subsequent nodule formation through AON (autoregulation of nodulation) pathway. In *L. japonicus, LjIPT3* gene was shown to be activated in the shoot phloem via the components of AON system, negatively affecting nodulation. However, in *M. truncatula*, the detailed analysis of *MtIPT*s expression, both in roots and shoots, in response to nodulation has not been performed yet, and the link between *IPT*s and AON has not been studied so far. In this study, we performed an extensive analysis of *MtIPT*s expression levels in different organs, focusing on the possible role of *MtIPT*s in nodule development. *MtIPT*s expression dynamics in inoculated roots suggest that besides its early established role in the nodule primordia development, cytokinin may be also important for later stages of nodulation. According to expression analysis, *MtIPT3, MtIPT4*, and *MtIPT5* are activated in the shoots in response to inoculation. Among these genes, *MtIPT3* is the only one the induction of which was not observed in leaves of the *sunn-3* mutant defective in CLV1-like kinase, the key component of AON, suggesting that *MtIPT3* is activated in the shoots in an AON-dependent manner. Taken together, our findings suggest that *MtIPT*s are involved in the nodule development at different stages, both locally in inoculated roots and systemically in shoots, where their expression can be activated in an AON-dependent manner.

## Introduction

Cytokinins (CKs) are involved in different aspects of nodule development, playing a dual role in nodulation, depending on the time and place of their action. The exogenous application of cytokinin to legume roots induces responses similar to those of rhizobial Nod-factor (NF), including cortical cell division and expression of early nodulin genes (Cooper and Long, 1994; Heckmann et al., 2011). Moreover, the gain-of-function mutation in *Lotus histidine kinase1* (*Lhk1*) cytokinin receptor gene causes the formation of spontaneous nodules in *Lotus japonicus* (Tirichine et al., 2007). The loss-of-function mutations in *Lhk1* gene or RNAi-mediated downregulation of its ortholog in *M. truncatula* leads to a dramatic reduction in the nodule formation (Gonzalez-Rizzo et al., 2006; Murray et al., 2007, respectively). All these data suggest that cytokinins have a positive influence positively on the nodule development. However, elevated cytokinin levels can also locally contribute to the negative regulation of nodulation. *ckx3* mutants that are defective in cytokinin oxidase/dehydrogenase gene, involved in cytokinin degradation, exhibit reduced nodulation and infection thread formation together with elevated root levels of tZ (trans-zeatin) and DHZ (dihydrozeatin) cytokinins (Reid et al., 2016). In addition, the *lhk1* mutant that is defective in a cytokinin receptor gene in *L. japonicus* display**s** epidermal hyperinfection, suggesting that CKs negatively regulate epidermal infection (Held et al., 2014). However, in the *lhk1-1 lhk1a-1 lhk3* triple mutant, the infection thread is unable to enter the root cortex, which indicates that CKs positively regulate the infection thread entry and growth in the cortex (Miri et al., 2016). Moreover, the ectopic expression of *Arabidopsis AtCKX* (pEPI:AtCKX3) gene in the root epidermis of *Medicago truncatula* results in increased nodule number, while the ectopic expression of *AtCKX3* (pCO:AtCKX3) in the root cortex is associated with decreased nodule number (Jardinaud et al., 2016). Thus CKs act both positively and negatively on the nodulation depending on the tissue: while CKs are essential for the nodule primordium initiation and the infection process in the root cortex, they negatively regulate the infection in the epidermis (for review see Gamas et al., 2017).

CKs biosynthesis is a multistep process. The first and rate-limiting step of CKs biosynthesis is catalyzed by the adenosine phosphate isopentenyl transferase (IPT). Nine *IPT* genes (*AtIPT1–AtIPT9*) have been identified in *Arabidopsis thaliana*, exhibiting different expression patterns both in the shoots and the roots (Takei et al., 2001; Miyawaki et al., 2004). Among them, seven *IPT* genes (*AtIPT1* and *AtIPT3–AtIPT8*) encode ATP/ADP-isopentenyltransferases that have been shown to isopentenylate ATP and ADP (Kakimoto, 2001). AtIPT2 and AtIPT9 are tRNA-isopentenyltransferases which are supposed to supply cis-zeatin-type cytokinins in *A. thaliana* (Miyawaki et al., 2004). The production of trans-zeatin (tZ) nucleotide is catalyzed by the cytochrome P450 monooxygenase (CYP735A family) via an hydroxylation of the isopentenyl adenine nucleotide (Takei et al., 2004). Eventually, the formation of active cytokinin nucleobases from nucleotides is mediated by the product of *LONELY GUY*s (*LOG*s) genes (Kurakawa et al., 2007). In legumes, several cytokinin biosynthesis genes are activated in response to rhizobium inoculation or NF treatment. For example, in *L. japonicus*, the expression of *IPT1/2/3/4* together with *CYP735A* and *LOG1/4* is increased in response to rhizobial inoculation (Chen et al., 2014; Reid et al., 2017). Furthermore, the overexpression of cytokinin biosynthesis pathway genes (*IPT3*, *LOG4*, and *CYP735A*) is sufficient to induce spontaneous nodule formation (Reid et al., 2017). In *M. truncatula*, the expression of some cytokinin biosynthesis genes occurs as early as 3 h after NF treatment (van Zeijl et al., 2015). Two *LOG* genes are activated in response to bacterial inoculation (Mortier et al., 2014). Moreover, RNA-seq analysis shows the expression of some *LOG* genes along with that of CYP735A1 gene (Medtr6g017325) in the epidermis of inoculated roots (Jardinaud et al., 2016). Activation of *IPT* and *LOG* genes has also been demonstrated in nodules of *Pisum sativum* (Azarakhsh et al., 2015; Dolgikh et al., 2017).

Furthermore, *LjIPT3* is activated in the shoot phloem via the components of the AON (autoregulation of nodulation) system which negatively affects nodulation (Sasaki et al., 2014). AON represents a systemic regulation controlling the number of root nodules; it involves long distance signaling and communication between the roots and the shoot (for review see Reid et al., 2011; Mortier et al., 2012; Soyano et al., 2014). AON was shown to be activated when the first nodule primordia are formed (Caetano-Anolles and Gresshoff, 1991; Li et al., 2009). One of its key components is the product of a CLV1-like (CLAVATA1-like) receptor kinase gene [*SUPER NUMERIC NODULES* (*SUNN*) in *M. truncatula* and *HYPER NODULATION ABERRANT ROOT FORMATION1* (*HAR1*) in *L. japonicus*], which acts in the shoot. Mutations in the CLV1-like kinase gene lead to a shoot-controlled supernodulating phenotype in different legume species (Krusell et al., 2002; Nishimura et al., 2002; Searle et al., 2003; Schnabel et al., 2005). According to the present model of AON (for review see Soyano et al., 2014), CLE-peptides produced in nodules are transported to the shoot, where they bind to the CLV receptor complex including CLV1-like kinase; consequently, the response to suppress excessive nodule formation on roots is triggered. A set of studies suggests that the CLV receptor complex may include several proteins. In *M. truncatula*, the proteins CORYNE (CRN) and CLAVATA 2 (CLV2) interact with SUNN (Crook et al., 2016), and a mutation in the *CLV2* gene also leads to a supernodulating phenotype in legume plants (Krusell et al., 2011). In *L. japonicus*, the *KLAVIER* gene, the defect of which is also characterized by a shoot-controlled supernodulating phenotype, encodes a receptor-like kinase, structurally unrelated to CLV1-like kinase, that was shown to interact with HAR1, suggesting that these proteins may form a receptor complex which would perceive CLE-peptides (Miyazawa et al., 2010). Among the factors acting downstream of CLV1-like kinase in the root, the product of the *TOO MUCH LOVE* (*TML*) gene, an F-box protein, was revealed; its mutation results in a root-controlled supernodulating phenotype (Takahara et al., 2013).

The exact molecular nature of the shoot-derived signal which inhibits nodulation is still not well understood. It was shown that the *Mtsunn* mutant had increased amount of auxin transported from the shoot to the root. This indicates that the activation of CLV1-like kinase in the shoot leads to a reduction of auxin transport to developing nodules, thereby to a reduction in nodulation (van Noorden et al., 2006). The activation of *LjIPT3* in the shoot phloem, downstream of CLV1-like kinase, suggests that the shoot-to-root transport of both auxin and cytokinin is targeted by CLV1-like kinase, so that in roots the auxin amount is reduced and the cytokinin level is increased to restrict subsequent nodule formation. Other hormones such as jasmonic acid (JA) have also been implicated in the shoot-to-root communication during AON (Kinkema and Gresshoff, 2008; Reid et al., 2012).

In our previous study we had showed that the orthologs of *LjIPT1* and *LjIPT3* genes in *M. truncatula*, *Medtr1g110590* (*MtIPT1*) and *Medtr1g072540* (*MtIPT3*) respectively, were also upregulated in developing nodules at 7–9 days after rhizobial inoculation (Azarakhsh et al., 2015). However, the detailed analysis of *MtIPT*s expression, both in the roots and the shoots, in response to nodulation has not been performed and the link between *IPT*s and AON has not been studied in *M. truncatula* so far. In this study, we estimated, both in the shoots and the roots, the expression levels of all *MtIPT*s identified in databases at different time points after rhizobial inoculation. Moreover, we analyzed the expression of *MtIPT*s in the *sunn-3* mutant to identify *MtIPT*s potentially involved in the systemic control of nodulation in a *SUNN*-dependent manner.

## Materials and Methods

### Plant Material, Bacterial Strains, and Growth Conditions

*Medicago truncatula* Gaertn. Jemalong wild-type A17 and *sunn-3* mutant plants were grown in the growth chambers (16 h/8 h day/night regime, 21°C, and 75% relative humidity). The seeds were surface-sterilized with concentrated sulphuric acid for 10 min and washed five to six times with sterile water. The seeds were transferred on 1% agar and were kept at 4°C for 24 h; they were germinated at room temperature in darkness for 48 h. For temporal expression analysis *M. truncatula* plants were grown in vermiculite-containing pots moistened with nitrogen-free Fahraeus medium (Fahraeus, 1957). Ten days after germination, individual plants were inoculated with 1 ml of a *Sinorhizobium meliloti* (strain Sm2011) culture (OD600-0.7). Infected sites of the root with developing nodules as well as the shoot (the first leaves, the second leaves) were harvested at different stages after rhizobial inoculation. Non-inoculated plants grown in the same conditions were used as control. In the temporal expression analysis of *MtIPT*s performed at different stages of nodule development [from 1 to 21 days after inoculation (dpi), see Supplementary Figure S2 for microscopy images of the nodule developmental stages], we used 3 control time points [non-inoculated plants (NI) at 3, 5, and 7 days]. To avoid harvesting lateral root primordia, only the segments between emerged lateral roots were collected. Nodules were obtained at different stages after inoculation from the infected sites of the roots. For the expression analysis in different organs, plants were grown under the same conditions as for the temporal expression analysis, and the first leaf, the second leaf, shoot apex, stem, root tip and cotyledons were harvested at 16 days after germination, i.e., 6 days after inoculation.

### Quantitative Reverse Transcription PCR (qRT-PCR) Analysis

Total RNA was isolated from the plant tissues with an RNeasy Plant Mini Kit (Qiagen, Germany) according to the manufacturer’s instructions. DNase treatment was done using Rapid Out DNA Removal Kit (Thermo Fisher Scientific, United States). The quality of the samples was controlled and quantified with a Nano Drop 2000c UV-Vis Spectrophotometer (Thermo Fisher Scientific, United States). cDNA synthesis was performed with equal amount of RNA for all the timepoints in each experiment (varying between 400 ng up to 1 μg of RNA in different experiments), using Revert Aid Reverse Transcriptase kit (Thermo Fisher Scientific, United States). To check DNase treatment efficacy, qRT-PCR analysis of control samples without reverse transcriptase was performed. The qRT-PCR experiments were done on a CFX-96 real-time PCR detection system with a C1000 thermal cycler (Bio-Rad Laboratories, United States). The detection was achieved using SYBR Green and Eva Green intercalating dyes (Bio-Rad Laboratories, United States). All qRT-PCR reactions were done in triplicate. Cycle threshold (Ct) values were obtained using CFX96 manager software, and the data were analyzed by the 2^-ΔΔ^*^C^*^t^ method (Livak and Schmittgen, 2001). The relative expression was normalized against the constitutively expressed actin 11 gene in *Medicago*. cDNA sequences were taken from the *M. truncatula* genome database Mt4.0v1. All primers (Supplementary Table S1) were designed using Vector NTI Advance 10 software (Thermo Fisher Scientific, United States), and were synthesized by Evrogen (Evrogen, Russia). The specificity of PCR amplification was confirmed based on dissociation curve (55–95°C). For each experiment, at least three independent biological repeats were performed. The materials for each biological repeat of the shoot or the root/nodule were taken from 4 plants.

### Computer Software and Statistical Methods

Multiple alignment of nucleotide sequences was performed using Clustal W algorithm (Thompson et al., 1994) in Vector NTI Advance 10 software (Thermo Fisher Scientific, United States). For phylogenetic analysis, nucleotide sequences were retrieved from phytozome^1^ for *M. truncatula* and *A. thaliana* and from Genbank NCBI database^2^ for *L. japonicus*. Sequences were aligned with the MEGA6 program using Clustal W and the phylogenetic tree was generated using Maximum Likelihood method based on Tamura-Nei model (Tamura and Nei, 1993; Hall, 2013) with 1000 bootstrap replicates.

One-way ANOVA, Kruskal and Wallis test, and Student’s *t*-test were used to compare the gene expression levels of different samples. The graphs indicate mean with 95% confidence interval. At least three independent biological repeats were done for each experiment. For each time point (inoculated or non-inoculated) and in each biological repeat, four plants were used.

## Results

### Identification of *Medicago truncatula IPT* Genes

**FIGURE 1:**
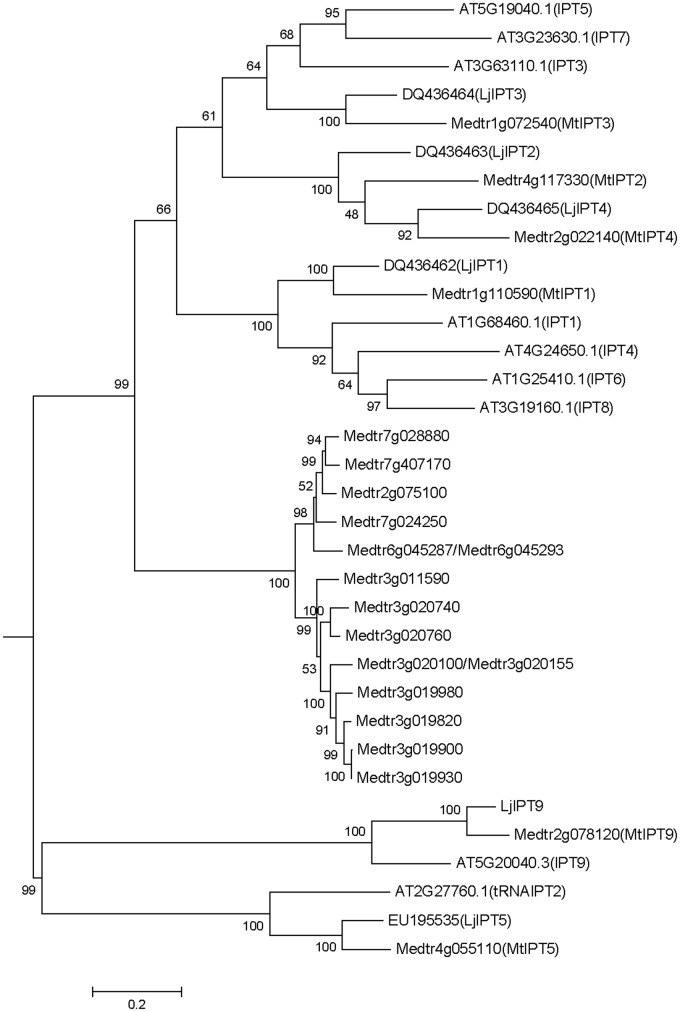
Phylogenetic tree for *Medicago truncatula*, *Arabidopsis thaliana*, and *Lotus japonicas IPT* genes constructed based on their nucleotide sequences. Tree was obtained using Maximum likelihood algorithm with 1000 bootstrap replicates. *Medicago* and *Arabidopsis* gene (locus) IDs and *Lotus* Genbank accession numbers are indicated on the tree.

*Medicago truncatula* genomic data (Mt4.0v1) contain 23 sequences annotated as isopentenyl transferases (IPT), two pairs of which are exactly the same sequences (*Medtr6g045287*/*Medtr6g045293* and *Medtr3g020100*/*Medtr3g020155*). Among the 21 non-repeated sequences, there are two sequences encoding truncated peptides (Medtr7g007180 and Medtr7g007190 with 57 and 59 amino acids, respectively). A phylogenetic tree was obtained using the nucleotide sequences of *IPT* genes of *M. truncatula, A. thaliana* and *L. japonicus* (**Figure 1**). According to the phylogenetic tree, *IPT* genes can be divided into five groups, one of which is unique for *M. truncatula*. This group contains 15 members that are highly similar in sequences with 52.6% identity and 95.45% consensus positions (Supplementary Figure S1). Other *M. truncatula IPT* genes are clustered with *A. thaliana* and *L. japonicus* genes into four groups. *MtIPT1,3,4* (*Medtr1g110590, Medtr1g072540*, and *Medtr2g022140*, respectively) have been identified previously (Azarakhsh et al., 2015), and they were named according to their nearest orthologs in *L. japonicus*. Similarly, here we refer to the other *M. truncatula* genes according to their near orthologs in *L. japonicus* and *A. thaliana*. *Medtr4g117330* is referred to as *MtIPT2*, *Medtr4g055110* as *MtIPT5* and *Medtr2g078120* as *MtIPT9*. Because the two latter genes are clustered with *AtIPT2*, *AtIPT9* and *LjIPT5*, *LjIPT9* which are all known to be tRNA IPTs (Kakimoto, 2001; Chen et al., 2014), *MtIPT5* and *MtIPT9* were annotated as tRNA isopentenyl transferases.

### Expression Pattern of *MtIPT* Genes

**FIGURE 2:**
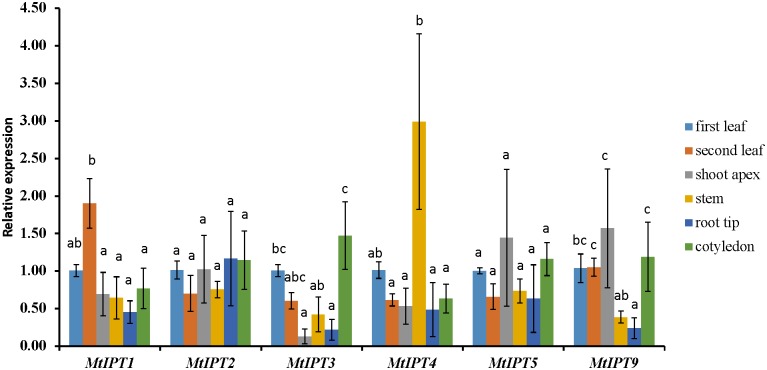
qRT-PCR expression analysis of *MtIPT*s in different organs. Samples are first leaf, second leaf, shoot apex, stem, root tip, and cotyledon. The first leaf for each gene was used as reference sample. The results are means ± 95% confidence intervals of three biological repeats. Different letters indicate significant differences in relative expression levels according to Kruskal and Wallis test (*P* < 0.05).

The expression levels of *MtIPT* genes were examined using qRT-PCR in different organs (the first and the second leaves, shoot apex, stem, root tips, and cotyledons) and the first leaf was used as the reference tissue. *MtIPT1* is expressed in all organs we tested with a slightly higher expression in the second leaf. As for *MtIPT2*, it is expressed in all analyzed organs at the comparable levels. *MtIPT3* exhibits a significantly lower expression in shoot apex and root tip than in the first leaf and cotyledons, and *MtIPT4* shows a higher expression in the stem. *MtIPT5* is expressed in all analyzed organs at the comparable levels, whereas *MtIPT9* has lower expression in the stem and in the root tip in comparison with other organs. (**Figure 2**). Most genes from the *M. truncatula* unique group show no expression in the different organs analyzed (no signal at all or Ct values close to water controls) except for *Medtr6g045287*, *Medtr7g028880*, and *Medtr7g407170*, which exhibited low expression levels in all analyzed organs (data not shown).

### Expression of *MtIPT* Genes in Response to Rhizobial Inoculation

To study the temporal expression of *MtIPT* genes during the nodule development, relative transcript levels were estimated at different timepoints after the rhizobial inoculation (days post inoculation, dpi) and compared to transcript levels of non-inoculated control roots (see Supplementary Figure S2 for microscopy images of the nodule developmental stages).

Previously, we had analyzed the expression dynamic of three *MtIPT*s (*MtIPT1, MtIPT3*, and *MtIPT4*) in response to the rhizobium inoculation (See Figure 5 from Azarakhsh et al., 2015). We had found that *MtIPT1* gene expression dramatically increased at 7 and 9 dpi, compared with non-inoculated control. Moreover, the expression level of *MtIPT3* was also increased during the nodulation. Although van Zeijl et al. (2015) had found activation of *MtIPT4* 3 h after NF treatment, we had not detected a significant *MtIPT4* expression upon nodule development (see Figures 5A–C from Azarakhsh et al., 2015).

**FIGURE 3:**
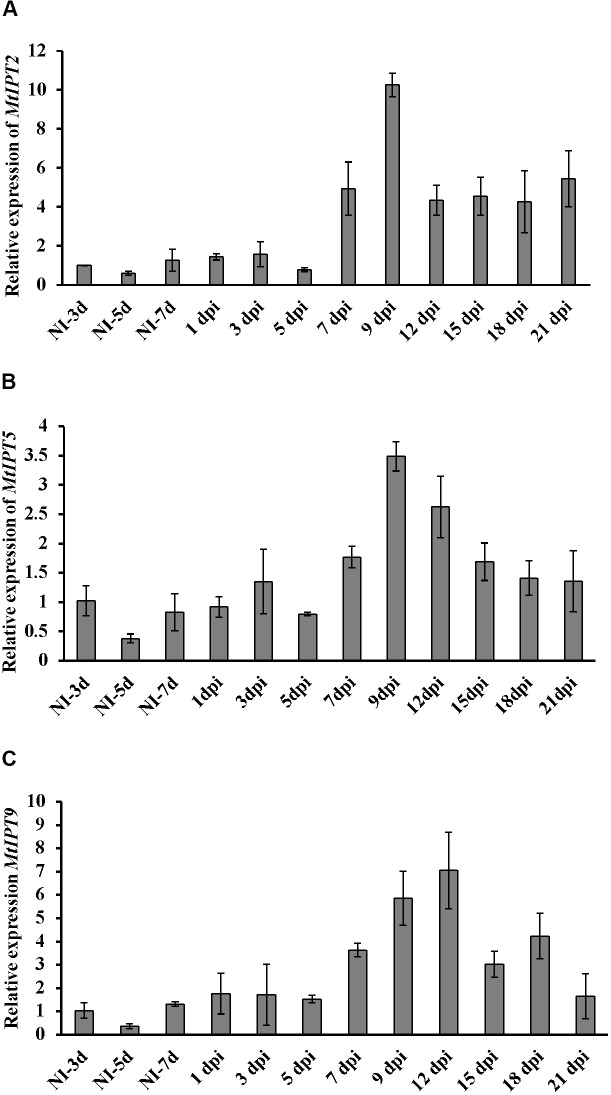
qRT-PCR expression analysis of *MtIPT2* **(A)***, MtIPT5* **(B)**, and *MtIPT9* **(C)** in non-inoculated roots (NI) and at different days post inoculation (dpi) in wild type plants (A17). The relative expression was normalized against constitutively expressed *M. truncatula* actin gene. Results are mean ± SEM of three technical repeat of one biological repeat, representative for three independent experiments.

Here, we found that the expression level of *MtIPT2* increased at 7 dpi, reached a 10-fold increase at 9 dpi in comparison to non-inoculated control (NI-3d), and remained high until the late stages of nodule development (up to 4-fold increase at 21 dpi). *MtIPT5* was slightly activated throughout nodule formation with a maximum of 3-fold increase at 9 dpi in comparison to non-inoculated control (NI-3d). Expression of *MtIPT9* was also increased up to 6–7 fold at 9–12 dpi (**Figure 3**). None of the genes from *M. truncatula* unique group were activated during nodule development (data not shown). According to the *M. truncatula* LCM-RNA-seq data^3^, *MtIPT1* and *MtIPT4* were expressed mostly in the nodule meristem, while *MtIPT2* shows higher expression in the meristem region, the distal and proximal infection zone (Supplementary Figure S3).

**FIGURE 4:**
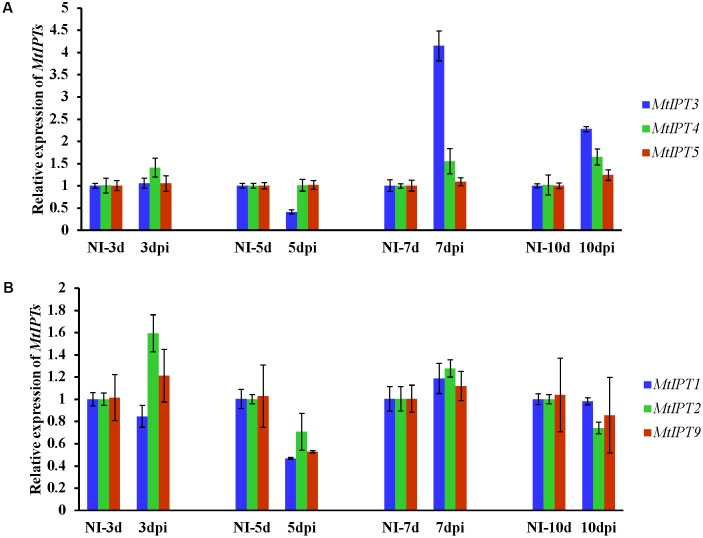
Relative expression of *MtIPT3*, *MtIPT4*, *MtIPT5* **(A)**, and *MtIPT1*, *MtIPT2*, *MtIPT9* **(B)** genes in the first leaf at different days post inoculation (3, 5, 7, and 10 dpi) in comparison with non-inoculated plants (NI). Results are mean ± SEM of three technical repeat of one representative biological repeat out of three biological repeats.

**FIGURE 5:**
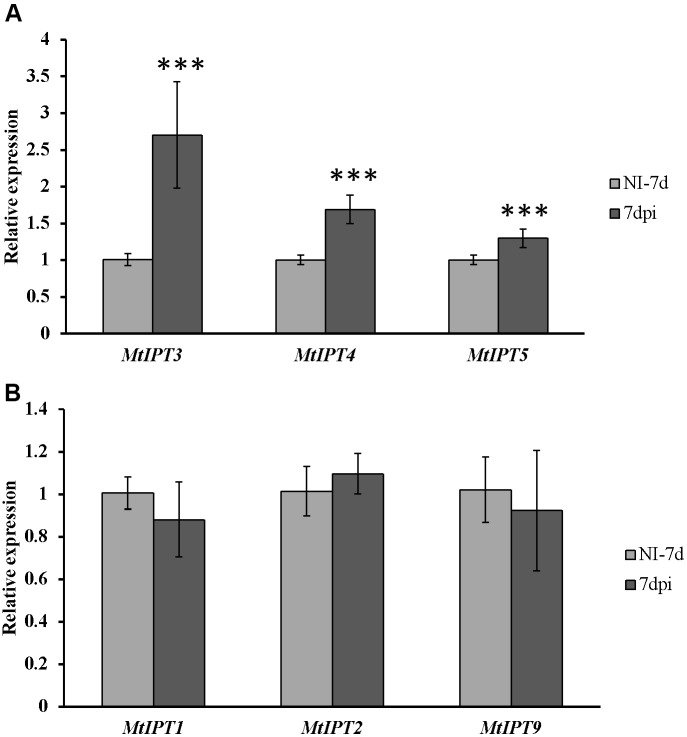
Relative expression of *MtIPT1-5* and *MtIPT9* in the first leaf of A17 plants at 7 dpi. The expression levels of *MtIPT3*, *MtIPT4*, and *MtIPT5* are increased **(A)**, while the expression levels of *MtIPT1*, *MtIPT2*, and *MtIPT9* do not changed significantly **(B)** in comparison with non-inoculated plants (NI). Asterisks indicate statistically significant differences compared with control (NI): ^∗∗∗^*P* < 0.001, ^∗∗^*P* < 0.01, ^∗^*P* < 0.05. Error bars indicate the 95% confidence interval of three biological repeats.

To find *MtIPT* genes potentially involved in AON, we estimated the expression of *MtIPT* genes in the shoot (the first leaf and the second leaf) at different days post inoculation (3, 5, 7, and 10 dpi). The expression levels of *MtIPT3* increased in the leaves in response to the rhizobial inoculation at 7 dpi (**Figures 4**, **5** and Supplementary Figure S4). However, among different biological repeats, the increased *MtIPT3* expression in the leaves relative to non**-**inoculated controls was also observed at other time points, in particular at 5 dpi (see Supplementary Figure S5). Nevertheless, it was at the 7 dpi timepoint that a statistically significant activation of *MtIPT3* was observed taking into account three biological repeats (**Figure 5**). Moreover, there was also a slight but statistically significant activation of *MtIPT4* and *MtIPT5* in the first leaves in response to inoculation, while such activation was not observed for *MtIPT1, MtIPT2*, and *MtIPT9* (**Figure 5**). Activation of *MtIPT4* and *MtIPT5* was confirmed in second leaves as well (Supplementary Figure S4). Activation of *MtIPT3, MtIPT4*, and *MtIPT5* expression in the leaves in response to inoculation may indicate the involvement of these genes in AON.

### Expression of *MtIPT* Genes in *sunn-3* Mutant

**FIGURE 6:**
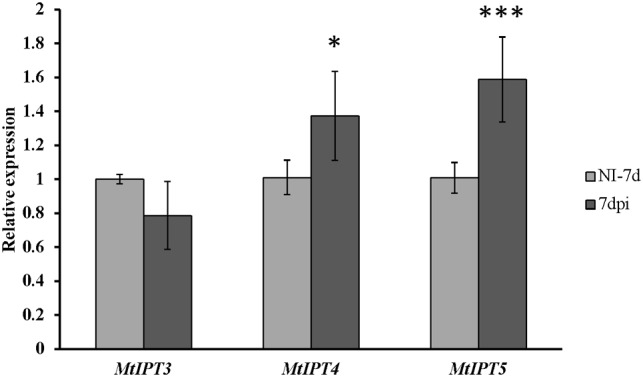
Relative expression of *MtIPT3*, *MtIPT4*, and *MtIPT5* in the first leaf of *sunn-3* mutant at 7 dpi in comparison with non-inoculated plants (NI). Asterisks indicate statistically significant differences compared with control (NI): ^∗∗∗^*P* < 0.001, ^∗∗^*P* < 0.01, ^∗^*P* < 0.05. Error bars indicate the 95% confidence interval of three biological repeats.

To address the question whether the activation of *MtIPT*s expression can be regulated by the key component of AON, i.e., the CLV1-like receptor, we estimated the expression levels of the *MtIPT* genes in both the shoots and the roots of the *sunn-3* supernodulating mutant. The expression levels of *MtIPT3*, *MtIPT4*, and *MtIPT5* genes that demonstrated activation in wild-type shoots at 7 dpi were estimated in the first and the second leaves of *sunn-3* mutants. In contrast to wild-type, *MtIPT3* did not show any activation in the *sunn-3* first and second leaves, while *MtIPT4* and *MtIPT5* exhibited increased expression at 7 dpi both in the wild-type and *sunn-3* leaves (**Figure 6** and Supplementary Figures S6, S7).

To find out if the activation of the *MtIPT* genes in the inoculated roots can be also regulated by CLV1-like kinase, we analyzed the expression level of *MtIPT* genes in the inoculated roots of *sunn-3* mutant at different stages after inoculation. According to qRT-PCR analysis, expression of *MtIPT1-3*, *MtIPT5*, and *MtIPT9* is activated in *sunn-3* mutant as well (Supplementary Figure S8). We did not find any changes in the general pattern of their expression in comparison with wild-type plants (for comparison see Figure 3 of this article and Figures 5A–C from Azarakhsh et al., 2015), which is consistent with an analogous experiment in *L. japonicus*. This suggests that *MtIPT3* expression in the shoots in response to inoculation may be regulated by CLV1-like kinase MtSUNN, while the activation of *MtIPT3* together with other *MtIPT* genes in the roots occurs independently of CLV1-like kinase MtSUNN.

## Discussion

In the entire *Medicago* genome Mt4.0v1, 23 sequences were annotated as isopentenyl transferases (IPT). Among them, according to the phylogenetic analysis, 15 sequences were clustered together as a separate group of sequences with a high degree of similarity, and two pairs of sequences from this group appeared to be exactly the same. This suggests that this group of *IPT*s sequences may represent new genes which appeared during evolution as a result of recent duplication events in the *Medicago* genome. However, according to the expression analysis, *IPT* genes from this group exhibited either no or very low level of expression in the *Medicago* tissues tested. Thus, a functional and more comprehensive phylogenetic analysis including *IPT* sequences from other legumes is required to elucidate the possible role of these evolutionary new *IPT* genes in legume development.

**FIGURE 7:**
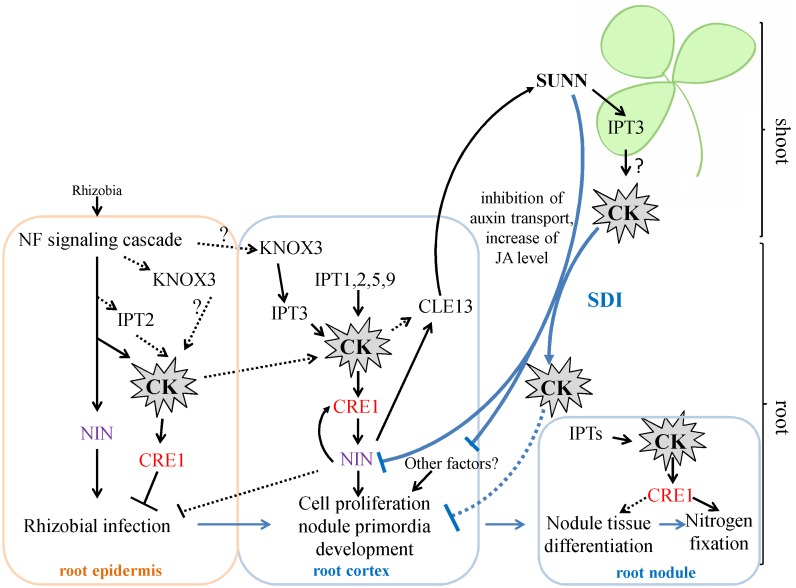
A scheme illustrating the role of *IPT*s and cytokinin in nodulation in *M. truncatula*. Cytokinin (CK) biosynthesis and response are activated both in root epidermis and in root cortex in response to rhizobial inoculation. The induction of *IPT* genes in root epidermis contributes to the negative effect of CK on rhizobial infection, while activation of *IPT* genes in root cortex is important for cortical cell divisions during nodule primordia development, where CK is known to be a positive regulator. The expression of *KNOX3*, a possible activator of cytokinin biosynthesis genes, is induced both in root epidermis and in nodule primordia. CK activates the expression of *NIN* in the root cortex, where NIN promotes *CRE1* expression. The *NIN* expressed in root cortical cells negatively regulates rhizobial infection. CK are important for nodule tissue differentiation and nodule meristem development, since *CRE1* was shown to be important for the transition between meristematic and cell differentiation/elongation zones in indeterminate nodules. Moreover, CK is also important for nitrogen fixation, since it was shown that CRE1 is necessary for this process. The expression of *IPT*s that was observed at later stages of nodule development in the present study is in agreement with this role of CK in subsequent nodule development and functioning. Being involved in AON, CK induces the *CLE13* gene. In its turn, MtCLE13 signal peptide activates SUNN receptor kinase in the shoot, triggering SDI that is transported to the roots where it inhibits subsequent nodulation. CK was shown to be the part of SDI, since in *L. japonicus LjIPT3* is activated in the shoot in HAR1-dependent manner, and shoot-derived cytokinin inhibits nodulation. *IPT3* activation in SUNN-dependent manner in the shoot is in agreement with these findings. In addition to CK, SDI involves the inhibition of auxin transport and the increase of JA level. SDI has multiple targets in the root, and NIN was shown to be one of them. CK – cytokinin. SDI – shoot-derived inhibitor.

Cytokinins have been previously shown to regulate different aspects of nodulation, including rhizobial infection during which CKs act as negative regulators and nodule primordia development and nitrogen fixation for which CKs act as positive regulators (Tirichine et al., 2007; Held et al., 2014; Boivin et al., 2016; Jardinaud et al., 2016). In *M. truncatula*, accumulation of cytokinins (iP: isopentenyladenine, iPR: isopentenyladenosine, tZ: trans-zeatin) was first observed at 3 h after NF exposure and this occurs in MtCCaMK-dependent manner. (van Zeijl et al., 2015). In our work, we did not study *IPT*s expression at very early stages nodule development, i.e., few hours after rhizobial infection. However, van Zeijl et al. (2015) reported that *Medtr2g022140* (*MtIPT4* according to our nomenclature) induced at 3 h after Nod factor treatment, therefore this *IPT* gene may contribute to cytokinin accumulation at early stages after rhizobial inoculation. Moreover, according to LCM-RNA-seq data, *MtIPT2* is activated in root epidermis 24 h after NF treatment (Jardinaud et al., 2016). Moreover, the expression of the *KNOX3* gene encoding homeodomain-containing TF that activates *IPT3* expression in developing nodules (Azarakhsh et al., 2015) was also induced in root epidermis according to LCM-RNA-seq data (Jardinaud et al., 2016). The early induction of cytokinin biosynthesis in root epidermis may contribute to its negative effect on rhizobial infection reported previously (Held et al., 2014, see **Figure 7**).

In pea, a two-stage increase in the content of CKs during nodule development was observed (Dolgikh et al., 2017). The accumulation of tZ and IP at 3 dpi can be associated with cortical cell division where CKs are known to be positive regulators, while the increase of tZ content at later stages suggests that CK also plays a role in subsequent stages of the nodule development and functioning (Dolgikh et al., 2017). The temporal expression dynamics of *MtIPT*s we observed are in line with this observation, suggesting that cytokinin biosynthesis also occurs at later stages of nodule development. According to our previously reported data, the expression of *MtIPT1* and *MtIPT3* increased in the inoculated roots reaching a maximum at 9 dpi (Azarakhsh et al., 2015). Here, we found that the expression of *MtIPT2*, *MtIPT9*, and *MtIPT5* genes were also induced during the nodule development, and the expression maximum of these genes is observed at 9–12 dpi, when the nodule primordia are completely developed.

The nodulation phenotype of the *Mtcre1* mutant defective in a cytokinin receptor suggested that besides having a crucial role in the early steps of nodule initiation, CKs regulate the subsequent stages of nodule development (Plet et al., 2011). The rare nodules which formed on *Mtcre1*-inoculated roots exhibit incomplete differentiation and have frequently multiple lobes, suggesting that a functional MtCRE1 may regulate the transition between meristematic and cell differentiation/elongation zones in the indeterminate nodules (Plet et al., 2011). Taking into account this finding, we suggest that *IPT*s expression that we observed after 7 dpi (at the stages when nodule differentiation occurs and the nodule meristem is being formed) may contribute to cytokinin involvement in the nodule differentiation and the meristem formation (see **Figure 7**).

Moreover, recently, it was shown that functional cytokinin receptor in *M. truncatula* is required for nitrogen fixation (Boivin et al., 2016). In *cre1* mutant showing reduced and delayed nodule formation, nitrogen fixation was decreased. Notably, the AHK4/CRE1 gene from *Arabidopsis* was able to complement nodule initiation, but not nitrogen fixation in the *cre1* mutant, indicating that legume-specific determinants encoded by MtCRE1 are likely required for nitrogen fixation activity (Boivin et al., 2016). This finding represents one more evidence that CKs act at later stages of nodule development and functioning.

In *L. japonicus*, it was shown that *LjIPT3* gene is activated in the shoot in response to rhizobial inoculation, and its activation is dependent on the CLV1-like kinase LjHAR1 (Sasaki et al., 2014). This increase in *LjIPT3* expression was observed at 3–5 dpi, which is consistent with the timing of AON induction determined previously for this legume in a split-root system (Suzuki et al., 2008). In our experiment we found that the expression of *MtIPT3*, the ortholog of *LjIPT3*, was activated in shoots at 7 dpi. The activation of *MtIPT3* in the leaves of inoculated plants is consistent with the data from phytozome database^4^. *MtIPT4* and *MtIPT5* were also slightly induced in shoots after rhizobial inoculation. Interestingly, the shoot *MtIPT3* activation in response to inoculation was absent in the *sunn-3* mutant defective in the CLV1-like kinase MtSUNN. These findings indicate that the ortholog of *LjIPT3* gene in *M. truncatula* is also activated in shoots in response to inoculation in a CLV1-like kinase-dependent manner, whereas the expression of *MtIPT3* together with other *MtIPT* genes in roots occurs independently of CLV1-like kinase. The activation of *MtIPT4* and *MtIPT5*, which was still observed in the *sunn-3* mutant in response to inoculation is unlikely to be regulated by MtSUNN, but instead it must be controlled by other regulatory pathways. The timing of AON induction in *Medicago* was determined to be 3 days after inoculation (Kassaw et al., 2015). In our experiments, the statistically significant activation of *MtIPT*s in leaves was observed at 7 dpi, although in one of three biological repeats the increased expression of *MtIPT*s in leaves was found at 5 dpi after rhizobial inoculation (Supplementary Figure S5). We suppose that temporal dynamics of nodule development may vary depending on experimental plant growth conditions, and the timing of AON activation may vary accordingly. Moreover, the expression of *MtCLE13*, a CLE-peptide that triggers AON, was shown to be activated as early as 3 h after NF treatment at a time-point preceding nodule primordium initiation (van Zeijl et al., 2015). According to Mortier et al. (2010), *MtCLE13* was significantly increased in inoculated roots at 4 dpi, and its expression level remained relatively high until 10 dpi, with a slightly pronounced maximum at 8 dpi. These findings allow us to propose that AON may comprise different levels of regulation, involving the perception of different signals. The shoot-derived signal (SDI) in AON also appeared to have a complex molecular nature. AON involves different hormones such as auxins, JA and cytokinins (van Noorden et al., 2006; Kinkema and Gresshoff, 2008; Sasaki et al., 2014), that may also inhibit nodulation at different levels via multiple targets. Among the later ones, the NIN transcription factor was shown to be targeted by AON in inoculated roots in a negative feed-back manner, where NIN also directly induces *CLE*s expression to trigger AON (Soyano et al., 2014). Together with CKs, NIN represents a crucial regulator of different aspects of nodulation that plays both positive and negative roles in nodulation. Along with the fact that NIN transcription factor is important both for rhizobial infection and nodule primodia development (Schauser et al., 1999; Marsh et al., 2007), *NIN* expression in the cortex was shown to inhibit rhizobial infection (Yoro et al., 2014). Moreover, *NIN* expression in the root cortex is activated by CK (Gonzalez-Rizzo et al., 2006; Plet et al., 2011), and NIN can promote *CRE1* expression (Vernié et al., 2015) (see **Figure 7**).

A large body of evidence indicates that CKs play a complex role in nodulation, mediating negative feedback regulatory mechanisms (Mortier et al., 2014; Sasaki et al., 2014). The exact molecular mechanisms underlying the dual effects of CKs on nodulation are still far from being understood. It is likely that CKs acting in a tight crosstalk with other hormones, such as auxins, may exert different effects on nodulation depending on the auxin concentration that is known to fluctuate through different stages of nodulation. Future studies should reveal the molecular pathways regulated by CKs and other hormones to elucidate their complex action during nodulation.

## Author Contributions

MA performed all the experiments on gene expression analysis. MA and ML analyzed the data and drafted the manuscript. ML and LL planned the experiments and supervised the research. All authors read and approved the manuscript.

## Conflict of Interest Statement

The authors declare that the research was conducted in the absence of any commercial or financial relationships that could be construed as a potential conflict of interest.

## Footnotes

1 https://phytozome.jgi.doe.gov/

2 https://www.ncbi.nlm.nih.gov/

3 https://iant.toulouse.inra.fr/symbimics/

4 https://phytozome.jgi.doe.gov/
