# Supplementary material for: Identification and Expression Analysis of Medicago truncatula Isopentenyl Transferase Genes (IPTs) Involved in Local and Systemic Control of Nodulation
Source: Front Plant Sci. 2018 Mar 9;9:304. doi: 10.3389/fpls.2018.00304 (PMC5855100; doi:10.3389/fpls.2018.00304)
Supplement: Supplementary file 1 [file Data_Sheet_1.PDF]

Supplementary

Figure S1. Alignment of coding sequences of *MtIPTs* from the 'unique' group with high similarity.

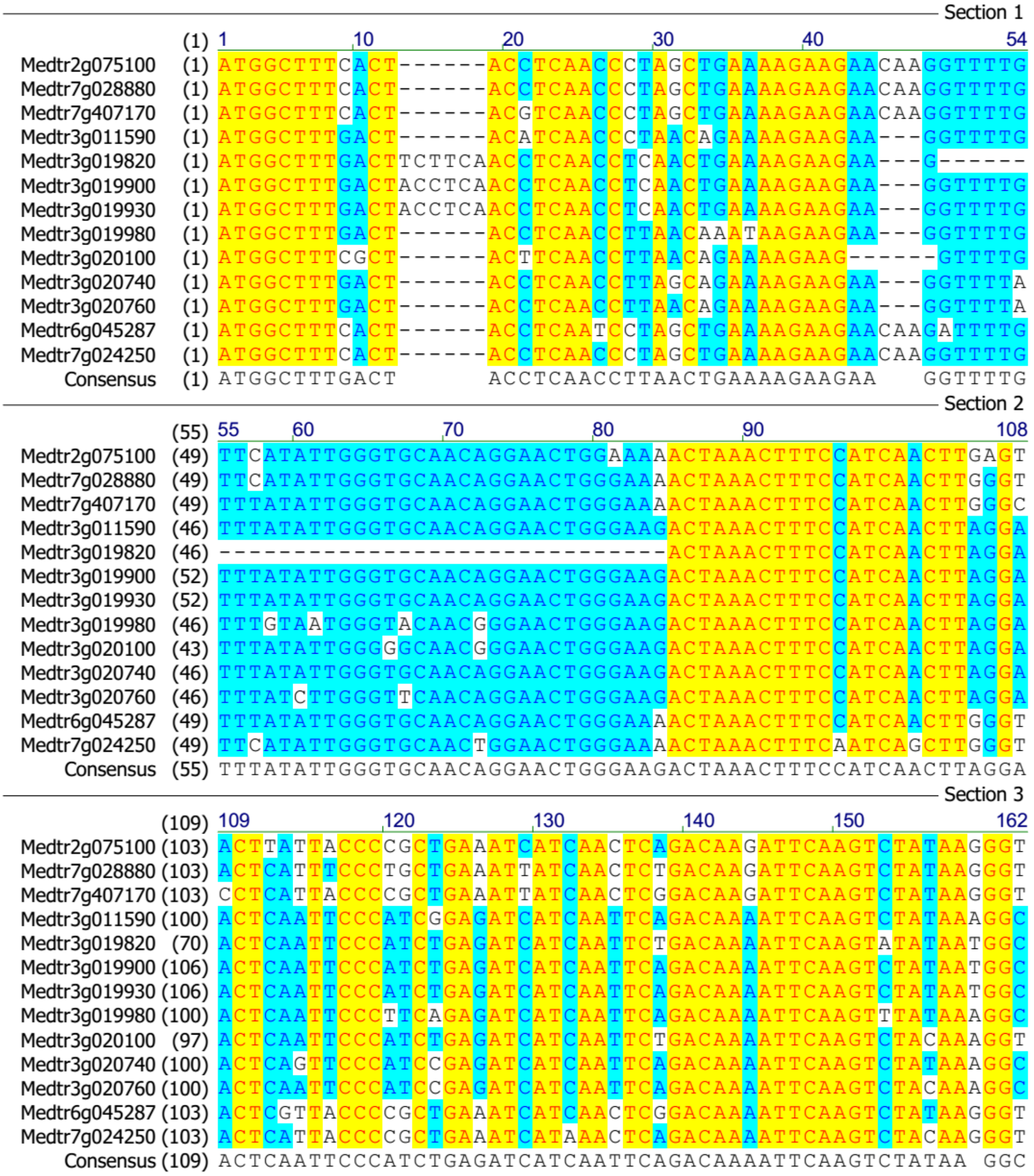

```
                                                                                  Section 4
              (163) 163       170               180       190       200              216
Medtr2g075100 (157) CTTGATATTGTCACAAATAAGGTACTGGAATCTGAACGTTGCTCGATTCCGCAT
Medtr7g028880 (157) CTTGATATTGCCACAAATAAGGTACCGGAATCTGAACGATGCTCGATTCCGCAT
Medtr7g407170 (157) CTTGATATCGTCACAAATAAGA-----------GGGCGTTACA-GATTCCGCAT
Medtr3g011590 (154) CTTGACATTGTCACCAATAAGGTACAAGAATCCGAACGTCATTCAATTCCACAT
Medtr3g019820 (124) CTTGACATTGTCACCAATAAGGTGCAGGAATCTGAACGTTGTTCAATTCCACAT
Medtr3g019900 (160) CTTGACATTGTCACCAATAAGGTGCAGGAATCTGAACGTTGTTCAATTCCACAT
Medtr3g019930 (160) CTTGACATTGTCACCAATAAGGTGCAGGAATCTGAACGTTGTTCAATTCCACAT
Medtr3g019980 (154) CTTGACATTGTCACCAATAAGGTACAGAAATCTGAACGTTGTTCAATTCCACAC
Medtr3g020100 (151) CTTGACATTGTCACCAATAAGGTACAAGAATCTGAACGTTGTTCAATTCCCCAT
Medtr3g020740 (154) CTTGACATTGTCACTAATAAGGTACAAGAATCTGAACGTTGTTCAATTCCTCAT
Medtr3g020760 (154) CTTGACATTGTCACCAATAAGGTACAGGAATCTGAACGTTGTTCAATTCCTCAT
Medtr6g045287 (157) CTTCATATTGTCACAAATAAGGTGCCGGAATCTGAACGTTGTTCGATTCCGCAT
Medtr7g024250 (157) CTTGATATTGTCACGAATAAGGTACCGGAATCTGAACGTTGTTCGATTCCGCAT
    Consensus (163) CTTGACATTGTCACCAATAAGGTACAGGAATCTGAACGTTGTTCAATTCC CAT
                                                                                  Section 5
              (217) 217          230       240       250       260              270
Medtr2g075100 (211) CATATATTAGGCATCATCGATGATCCTGAATATGATTTTACTATGAATGATTTC
Medtr7g028880 (211) CATATATTAGGCATCATCGATGATCCTGAATATGATTTTACTATGAATGATTTC
Medtr7g407170 (199) CACATATTAGGCATCATCAATGATCCTGAATATGACTTTACTATGTATGATTTC
Medtr3g011590 (208) CATATCTTAGGCATCATTGACGATCCCGAATATGATTTCACCGTGGATGATTTT
Medtr3g019820 (178) CATATATTAGGCATCATCGATGATCCTGAATATGATTTCACTATGGATGACTTT
Medtr3g019900 (214) CATATACTAGGCATCATCGATGATCCTGAATATGATTTCACTATGGATGACTTT
Medtr3g019930 (214) CATATACTAGGCATCATCGATGATCCTGAATATGATTTCACTATGGATGACTTT
Medtr3g019980 (208) CATATATTAGGCATAATCGATGATCCTGATTATGATTTCACTATGGATGATTTT
Medtr3g020100 (205) CATATATTAGGCATCATTGATGATCCTGAATATGATTTCACTATGGATGAGTTT
Medtr3g020740 (208) CATCTATTAGGCATCATTGATGATCCTGAATATGATTTCACTGTGGATGAGTTT
Medtr3g020760 (208) CATATATTAGGCATCATTGATGATCCTGAATATGATTTCACTGTGGATGATTTT
Medtr6g045287 (211) CACTTATTAGGCATCATCGATGATCCTGAATATGATTTTACTATGAACGATTTT
Medtr7g024250 (211) CACTTATTAGGCATCATCGATGATCCTGAATATGATTTTACTATGAATGATTTT
    Consensus (217) CATATATTAGGCATCATCGATGATCCTGAATATGATTTCACTATGGATGATTTT
                                                                                  Section 6
              (271) 271        280       290       300       310              324
Medtr2g075100 (265) TGCAAGCATGTGCTTGAATCCTTAGATCTAATAATTGGCAATGGACACCTACCT
Medtr7g028880 (265) TGCAACCACGTGCTTGAATCCTTAGATCTGATAACTGGCAAAGGACACCTACCT
Medtr7g407170 (253) TGCAACCACGTGGTTGAATCCCTAGATCTCATAATTGGCAATGGACACCTACCT
Medtr3g011590 (262) TGCAATCACGTGCTTGAAGCTTTAAATCTCATAACTGAAAATGGACACCTACCT
Medtr3g019820 (232) TGCAAGCACGTGCTTGAAGCTTTAGATCTCATAACTCAAAATGGACACCTACCT
Medtr3g019900 (268) CGCAAGCACGTGCTTGAAGCTTTAGATCTCATAACTCAAAATGAACACCTACCA
Medtr3g019930 (268) CGCAAGCACGTGCTTGAAGCTTTAGATCTCATAACTCAAAATGAACACCTACCA
Medtr3g019980 (262) TGCAAGCACGTGCTTGAGGCTTTAGACCTCATAACTCAAAATGGACACCTACCT
Medtr3g020100 (259) TGCAAGCACGTGCTTGAAGCTTTAGACCTCATAATTGAAAATGGACACCTACCT
Medtr3g020740 (262) TGCAAGCACGTTCTTGAAGCTTTGGCTCTTATAATTGCAAATGGACGCCTGCCT
Medtr3g020760 (262) TGCAAGCACGTGCTTGAAGCTTTAGATCTTATAATTGAAAATGGACACCTACCT
Medtr6g045287 (265) TGCAAGAACGTGCTTGAATCCATAGATCTCATAATTGGCAATGGACGCCTACCT
Medtr7g024250 (265) TGCAAACATGTGCTTGAATCCCTAGATCTGATAATTGGCAATGAGCACCTACCT
    Consensus (271) TGCAAGCACGTGCTTGAAGCTTTAGATCTCATAATTGAAAATGGACACCTACCT
```

```
                                                                   Section 7
           (325) 325 330       340       350       360               378
Medtr2g075100 (319) ATTATTGTAGGAGGGTCCAATTCTTATCTAAGAAAATTAGTTGAGGACCCAACC
Medtr7g028880 (319) ATTATTGTAGGAGGGTCCAATTCTTATCTAAAAAAATTAGTTGAGGACCCAGCC
Medtr7g407170 (307) ATTATTGTAGGAGGGTCCAATTCTTATCTAAAAAAATTAGTTGAGGACCCAGCC
Medtr3g011590 (316) ATTATTGTAGGAGGGTCAAATTCTTACCTAAAAAAATTAATTGAGGACCCAACC
Medtr3g019820 (286) ATTATTGTAGGAGGGTCAAATTCTTACCTTAAAAAACTACTCGAGGACCCAACT
Medtr3g019900 (322) ATTATTGTAGGAGGGTCGAATTCTTACCTTAAAAAACTACTCGAGGACCCAACT
Medtr3g019930 (322) ATTATTGTAGGAGGGTCGAATTCTTACCTTAAAAAACTACTCGAGGACCCAACT
Medtr3g019980 (316) ATTATTGTAGGAGGGTCAAATTCTTATCTTAAAAAATTACTCGAGGATCCAACT
Medtr3g020100 (313) ATTATTGTAGGAGGGTCAAATTCTTACCTTAAAAAATTACTCGAGGACCCAACT
Medtr3g020740 (316) ATCATTGTAGGAGGGTCAAATTCTTACCTAAAAAAATTAGTTGAGGACCCATCC
Medtr3g020760 (316) ATTATTGTAGGAGGGTCAAATTCTTACCTAAAAAAATTAGTTGACGACCCAGTC
Medtr6g045287 (319) ATTATTGTAGGAGGGTCCAATTCTTATATAAAAAAATTGGTTGAAGAGCCAACC
Medtr7g024250 (319) ATTATTGTAGGAGGGTCCAATTCTTATCTTAAAAAATTAGTTGAGGACCCATCC
    Consensus (325) ATTATTGTAGGAGGGTC AATTCTTACCTAAAAAAATTAGTTGAGGACCCAACC
                                                                   Section 8
           (379) 379        390       400       410       420        432
Medtr2g075100 (373) ATTGCATTTCTTTCAAAATATGATTGTTGTTTCATCTGGGTAGATGTGTCTTTG
Medtr7g028880 (373) ATTTCATTTCTTTCAAAATATGATTGTTGTTTCATTTGGGTAGACGTGTCTTTG
Medtr7g407170 (361) ATTTCATTTCTTTCAAAATATGAATGTTGTTTCATTTGGGTAGACGTGTCTTTG
Medtr3g011590 (370) ATTGCATTTCATTCTAAATATGACTGTTGTTTTATTTGGGTCGACGTGTCTTTG
Medtr3g019820 (340) AATGCATTTCATTCAAAATACGATTGCTGTTTTATTTGGCTCGATGTGTCTTTA
Medtr3g019900 (376) AATGCATTTCATTCAAAATATGATTGCTGTTTTATTTGGCTCGATGTGTCTTTA
Medtr3g019930 (376) AATGCATTTCATTCAAAATATGATTGCTGTTTTATTTGGCTCGATGTGTCTTTA
Medtr3g019980 (370) ATTGCATTTCATTCAAAATATGATTGCTCTTTTATTTGGCTAGATGTGTCTTTG
Medtr3g020100 (367) ATTGCATTTCGTTCAAAATATGATTGCTGTTTTATTTGGTTAGATGTGTCTTTG
Medtr3g020740 (370) ATTGCATTTCATTCAACATATGATTGTTCTTTTGTTTGGCTTGACGTATCTTTG
Medtr3g020760 (370) ATTGCATTTCATTCAAAATATGATTGTTGTTTTATTTGGGTCGACGTGTCTCTG
Medtr6g045287 (373) ATTGCTTTTCTTTCAAAATATGACTGTTTTTTCATTTGGGTAGATGTGTCTTTG
Medtr7g024250 (373) ATTGCATTTTTTTCAAAATATGATTGTTGCTTCATTTGGATCGACGTGTCTTTG
    Consensus (379) ATTGCATTTCATTCAAAATATGATTGTTGTTTTATTTGG T GATGTGTCTTTG
                                                                   Section 9
           (433) 433    440       450       460       470            486
Medtr2g075100 (427) CCTACTCTGTTTAAATATGTAGGCAAAAGAGTGGATGAAATGGTTGAGGGTGGG
Medtr7g028880 (427) CCTACTCTCTATCAATATGTAGGCAAAAGAGTTGATGAAATGGTCCAGGCAGGG
Medtr7g407170 (415) CCTACTCTATATCAATATGTAGGAAAAAGAGTTGATGAAATGGTTCAGACAGGG
Medtr3g011590 (424) CCTATTCTATGTCCATACTTAGACAAAAGAGTTGATGAAATGGTTGAGTCAGGG
Medtr3g019820 (394) CCTATTTTGTTTCCATATTTGGACAAAAGAGTTGATGAAATGGTTGCGGCAGGG
Medtr3g019900 (430) CCTATTTTGTTTCCATATTTGGACAAAAGAGTTGGTGAAATGGTTGCGGCAGGG
Medtr3g019930 (430) CCTATTTTGTTTCCATATTTGGACAAAAGAGTTGATGAAATGGTTGCGGCAGGG
Medtr3g019980 (424) CCTATTCTGTTTCCATATTTGGACAAAAGAGTTGATGAAATGGTTGCGGTAGGG
Medtr3g020100 (421) CCTATTCTTTTTCCATATTTGGACAAAAGAGTTGATGAAATGGTTCAGGCAGGG
Medtr3g020740 (424) CCTATTCTGTTTCCATATTTGGACAAAAGAGTTGATGAGATGGTTGGGGCAGGG
Medtr3g020760 (424) CCTATTCTCTTTCCATATTTGGACAAAAGAGTGGATGAGATGGTTGAAGCAGGG
Medtr6g045287 (427) CCTACTCTATTTCAATATGTAGGGAAAAGAGTTGATGAAATGGTTGAGTCAGGG
Medtr7g024250 (427) CCTACTCTGTTTCAATATGTAGGCAAAAGAGTCGATGAAATGGTAGAGGCAGGG
    Consensus (433) CCTATTCTGTTTCCATATTTGGACAAAAGAGTTGATGAAATGGTTGAGGCAGGG
```

```
Section 10
                    487          500       510       520       530      540
      (487)
Medtr2g075100 (481) ATGGTTGATGAGATTCGAGAATATTTTGTACCTGGGGCAGACAACACAAAGGGA
Medtr7g028880 (481) ATGGTTGATGAGATTCGAGAATATTTTGTACCTGGGGCAGATAACACAAAGGGA
Medtr7g407170 (469) ATGATTGATGAGATTCGAGAATATTTTGTACCTGGGGCAGACAACACAAAGGGA
Medtr3g011590 (478) ATGGTAGATGAGATAAGAGACTTCTTTGTACCTGGAGCAGATTACACAAAAGGA
Medtr3g019820 (448) ATGGTAGATGAGATAAGAGACTTTTTTGTACTTGGAGCAGATAACACAAAAGGA
Medtr3g019900 (484) ATGGTAGATGAGATAAGAGACTTTTTTGTACCTGGAGCAGATAATACAAAAGGA
Medtr3g019930 (484) ATGGTAGATGAGATAAGAGACTTTTTTGTACCTGGAGCAGATAATACAAAAGGA
Medtr3g019980 (478) ATGGTAGATGAGATAAGAGACTTTTTTGTACCTGGAGCAGATAACACAAAAGGA
Medtr3g020100 (475) ATGGTAGATGAGATAAGGGATTTCTTTGTACCTGGTGCGGATAACACAAAAGGA
Medtr3g020740 (478) ATGGTAGATGAGATAAGAGAATTTTTTGTGCCTGGAGCAGACAACACAAAAGGA
Medtr3g020760 (478) ATGGTAGATGAGATAAGAGAATTTTTTGTACCTGGAGCAGACAACACAAAAGGA
Medtr6g045287 (481) ATGGTTGATGAGATTCGAGAATATTATGCACCTGGGGCAGACAACTCAAAGGGA
Medtr7g024250 (481) ATGGTCGATGAGATTCGAGAATATTTTGTACCTAGGGCGGACAACACAAAGGGA
    Consensus (487) ATGGTAGATGAGATAAGAGAATTTTTTGTACCTGGAGCAGATAACACAAAAGGA

Section 11
                    541      550       560       570       580          594
      (541)
Medtr2g075100 (535) ATTAGAAGGGCTATTGGGGTTCCTGAGCTTGATTCTTTTTTCGCGATAGAAAAG
Medtr7g028880 (535) ATTAGAAGGGCTATTGGGGTTCCTGAGCTCGATTCTTTTTTTGCGATAGAAAAG
Medtr7g407170 (523) ATTAGAAGGGCTATTGGGGTTCCTGAGCTTGATTCTTTTTTTGCGATTGAAAAG
Medtr3g011590 (532) ATTAGAAGGGCTATTGGGGTTCCTGAGCTTCATTCTTATTTTGAGATAGAAAAG
Medtr3g019820 (502) ATAAGAAGGGCAATTGGGGTTCCTGAGCTCGACTCTTATTTTGAAATGGAAATG
Medtr3g019900 (538) ATAAGAAGGGCAATTGGGGTTCCTGAGCTCGACTCTTATTTTGAAATGGAAATG
Medtr3g019930 (538) ATAAGAAGGGCAATTGGGGTTCCTGAGCTCGACTCTTATTTTGAAATGGAAATG
Medtr3g019980 (532) ATTAGAAGGGCAATTGGGGTTCCTGAGCTCGATTCTTATTTTGAAATGGAAATG
Medtr3g020100 (529) ATTAGAAGGGCTATTGGGGTTTCTGAACTTGATTCTTATTTTGAAATAGAAATG
Medtr3g020740 (532) ATTAGAAGGGCTATCGGGGTGCCAGAGCTTGATTCTTACTTTGAAATGGAAATG
Medtr3g020760 (532) ATTAGAAGGGCTATTGGGGTTCCAGAGCTTGATTCTTACTTTGAAATAGAAATG
Medtr6g045287 (535) ATTAGAAGGGCTATTGGGGTTCCTGAGCTTGATTCTTTTTTTCAAATAGAAAAG
Medtr7g024250 (535) ATTAGAAGGGCTATTGGGGTTCCTGAGCTTAATTCTTTTTTTGAGATAGAAAAG
    Consensus (541) ATTAGAAGGGCTATTGGGGTTCCTGAGCTTGATTCTTATTTTGAAATAGAAATG

Section 12
                    595  600       610       620       630              648
      (595)
Medtr2g075100 (589) AAAAGTGGCATTGATGATGCTATAAAGGAAATGATACTTAAGAAAGCTATTGAA
Medtr7g028880 (589) AAAAGTTGCATTGGTGATGCTATAAAGGAAAAGATACTGAAGGAAGCTATTGAA
Medtr7g407170 (577) AAAAGTGGCATTGATGATGCTATAAAGGAAAATATACTGAAGGAAGCTATTGAA
Medtr3g011590 (586) AAAGAATGTATTGATGATGCTCAAAAAGAAAAGATACTAAAGGAAGCTATTACA
Medtr3g019820 (556) AAAAAAGGTATAGATGATGCTGAAAAGGAAAAGATATTAAAGGAATCGATTAGA
Medtr3g019900 (592) AAAAAAGGTATAGATGATGTTGAAAAGGAAAAGATATTAAAGGAATCGATTAGA
Medtr3g019930 (592) AAAAAAGGTATAGATGATGTTGAAAAGGAAAAGATATTAAAGGAATCGATTAGA
Medtr3g019980 (586) AAAAAAGGTGTAGATGATGCTCAAAAGGAAAAGATATTACAAGAATCGATTAGA
Medtr3g020100 (583) AAAAAAGGTATAGATGATGCTCAGAAGGAAAAGATACTAAAGGAAGCAATTAGA
Medtr3g020740 (586) AAAAAAGGTGTAGATGATGCTGAAAAGGAAAAGATACTGAAGGAAGCGATTAGA
Medtr3g020760 (586) AAAAAAGGTATAGATGATGCTGAAAAGGAAAAGATACTAAAGGAAGCGATTAGA
Medtr6g045287 (589) AAAAATGACATTGATGATGCTCAAAAGGAAAAGATATTGGCCGAAGCTATCAGA
Medtr7g024250 (589) AAAAATGGCATTGACGATGCTCAAAAGGAGAAGATTCTGAAGGAAGCTATTGGA
    Consensus (595) AAAAAAGGTATAGATGATGCT AAAAGGAAAAGATACTAAAGGAAGC ATTAGA
```

```
                                                                          Section 13
         (649) 649        660       670       680       690          702
Medtr2g075100 (643) GAAACCAAACAAAACACTTGCATACTGGCTAAAAATCAACTCTCGAAGATCCAG
Medtr7g028880 (643) AACACCAAACAGAACACTTGCATACTGGCTAAAAATCAACTCTCGAAGATCCAG
Medtr7g407170 (631) AAAACCAAACAGAACACTTGCATACTAGCTAAAAATCAACTCTCGAAGATCCAG
Medtr3g011590 (640) AAAACCAAACAAAATACTTTCATATTGGCTGAAAATCAACTCTCGAAGATCCGA
Medtr3g019820 (610) AAAACCAAACAAAACACCTTCATATTGGCTGAAAATCAATTGTCGAAGATCCAA
Medtr3g019900 (646) AAAACAAAACAAAACACCTTCATATTGGCTGAAAATCAAGTGTCGAAGATCCAA
Medtr3g019930 (646) AAAACAAAACAAAACACCTTCATATTGGCTGAAAATCAAGTGTCGAAGATCCAA
Medtr3g019980 (640) AAAACCAAACAAAACACCTTCATATTGGCTGAAAATCAATTGTCAAAGATCCAA
Medtr3g020100 (637) AAAACCAAACAAAACACCTTCATATTGGCTGAAAATCAATTGTCAAAAATCCAA
Medtr3g020740 (640) AAAACCAAACAAAACACCTTCATATTGGCTGAAAACCAACACTTGAAGATCCAA
Medtr3g020760 (640) AAAACGAAACAAAACACCTTCAAATTGGCTGAAAATCAACACTCGAAGATCCAA
Medtr6g045287 (643) AAAACCAAACAGAACACTTGCATATTGGTTCAAAATCAACTCTTGAAGATCAAG
Medtr7g024250 (643) AAAACGAAACATAACACTTGCATATTGGTTGAAAATCAACTCTTGAAGATCCAT
    Consensus (649) AAAACCAAACAAAACACCTTCATATTGGCTGAAAATCAACTCTCGAAGATCCAA
                                                                          Section 14
         (703) 703    710       720       730       740            756
Medtr2g075100 (697) AACATGACTCATATGCTTGAATCGATGGTGTATAAAATTGATTCTACGGAAGTC
Medtr7g028880 (697) AACATGGCTCGTATGCTCGGATCGATGGTGTACAAAATTGATTCAACTGAAGTC
Medtr7g407170 (685) AACATGGCTCATATGCTTGGATCGATGGTGTACAAAATTGATTCTACGGAAGTC
Medtr3g011590 (694) AATATGGCTGATAAGCTTGGATCAATGATAAACAATATAAATTCTACAGAAGTC
Medtr3g019820 (664) AATATGGCTGATACCCTTGGATCGATGATAATCAAAATTGATTCTACAGAAGTC
Medtr3g019900 (700) AATATGGCTGATACGCTTGGATTGATGATAAACAAAATTAATTCTACAGAAGTC
Medtr3g019930 (700) AATATGGCTGATACGCTTGGATTGATGATAAACAAAATTAATTCTACAGAAGTC
Medtr3g019980 (694) AATATGGCTGCTAAGTTTGGATCGATGATAAACAAAATTGATTCTACAGAAGTC
Medtr3g020100 (691) AATATGTCGGATAAGCTTGGATCGATGGTAAAGAAAATTAATTCTACAAAAGTC
Medtr3g020740 (694) AATATGGCTAATAAGCTTGGATCGATGATTAACAAAATTAATTCTACAAAAGTC
Medtr3g020760 (694) AATATGGCCGATAAGCTTGGATCGATGATTAAAAAAATTAATTCTACAGAAGTC
Medtr6g045287 (697) AATATGGCTCAAATCCTTGGGTCGATGGTATACAAGATTGATTCTACGGAAGTC
Medtr7g024250 (697) AACATGGCTGATATGTTTGGATCAATGGTGTATAGAATTGATTCTACGAAAGTC
    Consensus (703) AATATGGCTGATA GCTTGGATCGATGATAAACAAAATTGATTCTACAGAAGTC
                                                                          Section 15
         (757) 757          770       780       790       800      810
Medtr2g075100 (751) TTTGAGGCCCTTTTAAGGGGAGAAGATTATAAACATTTGCATCAAGAGATTGTG
Medtr7g028880 (751) TTTGAGGCCCTTTTAAGGGGAGAAGATTATAAACATGTGCATCAAGAGAGCGTG
Medtr7g407170 (739) TTTGAGACCCTTTTAAGGGGAGAAGATTATAAACACTTGCATCAAGAGATTGTG
Medtr3g011590 (748) TTTGAGGCCATTCTAAGGGGAGAAGATTATCATCATTTGCATCAAGAGATTGTG
Medtr3g019820 (718) TTTAAAGCCATTTTAAGGGGTGAAGATTATCAAAAATTGCATCATGAGATTGTG
Medtr3g019900 (754) TTTGAAGCCATTTTAAGAGGTGAAGATTATCAAAAATTGCATCAAGAGATTGTG
Medtr3g019930 (754) TTTGAAGCCATTTTAAGAGGTGAAGATTATCAAAAATTGCATCAAGAGATTGTG
Medtr3g019980 (748) TTTGAAGCTATTTTAAGGGGTGAAGATTATCAACATTTGCATCAAGAGATTGTG
Medtr3g020100 (745) TTTGAAGCCATTTTAAGGGGTGAAAATTATCAAGATTTGCATCAAGAAATTGTG
Medtr3g020740 (748) TTTGAGGCCATTCTAAGGGGGGAAGACTATCAACATCTGTATCAAGAGATTGTA
Medtr3g020760 (748) TTTGAGGTCATTCTAAGGGGGGAAGACTATCAACATTTGTATCAAGATATTGTA
Medtr6g045287 (751) TTTGAGGCCCTTCTGAAGGGAGAAGATTATAAACATTTGCATCAAGAGAATGTA
Medtr7g024250 (751) TTTGAGGCCCTTTTAAGGGGAGAAGATTATAAACATTTGCATCAAGAGATTGTG
    Consensus (757) TTTGAGGCCATTTTAAGGGG GAAGATTATCAACATTTGCATCAAGAGATTGTG
```

```
                                                                         Section 16
            (811) 811      820       830       840       850           864
Medtr2g075100 (805) ATTAAACCAAGCAAGGAGATAGTGAAGAGATTCCTAGAGGAGACGACTGATGAA
Medtr7g028880 (805) ATTAAGCCAAGCAAGGAGATAGTGAAGAGATTCCTGGAGGAGACGACTGACGAA
Medtr7g407170 (793) ATTAAGCCAAGCAAGGAGATAGTGAAGAGATTCCTAGAGGAGACGACTGATGGA
Medtr3g011590 (802) ATTAATCCAAGTATGAAGATAGTGAAGAGATTTCTAGAGGAGACAAGCCCTATA
Medtr3g019820 (772) ATTAAGCCAAGCATGAAAATAGTGAAGAGATTTCTAGAGGGGACAAGCTATGGA
Medtr3g019900 (808) ATTAAGCCAAGCATGAAAATAGTGAAGAGATTCCTAGAGGAGACAAGCCATGGA
Medtr3g019930 (808) ATTAAGCCAAGCATGAAAATAGTGAAGAGATTCCTAGAGGAGACAAGCCATGGA
Medtr3g019980 (802) ATTAAGCCAAGCATGAAAATAGTAAAGAGATTCCTAGAGGGGACAAGCCATGGA
Medtr3g020100 (799) ATTAAGCCAAGCATGAAAATAGTAAAGAGATTTTTAGAGGAGACAAGCCATGGA
Medtr3g020740 (802) ATTAAACCAAGCATGAAGATAGTGAAGAGATTTCTGGAGGAGACAAGCCCTAGA
Medtr3g020760 (802) ATTAAGCCAAGCATGAAGATAGTGAAGAGATTTCTTGAGGAGACAAACCCCAGA
Medtr6g045287 (805) GTTAAGCCAAGCATAGAGATAGTGAAAAGATTCCTAGAGGAGACACCTGTTGGA
Medtr7g024250 (805) ATTAAGCCAAGCAAGGAGATAGTGAAGAGATTCCTAGAGGAGACGACTGATGGA
    Consensus (811) ATTAAGCCAAGCATGAAGATAGTGAAGAGATTCCTAGAGGAGACAAGCCATGGA
                                                                         Section 17
            (865) 865  870       880       890       900               918
Medtr2g075100 (859) TTTGGATATGAAAAATATTCAAATGATAATGTGAAACATGCATCCAATGGTGTT
Medtr7g028880 (859) TTTGGATATGAAAAATATTCAAATGAAAATGGGAAACATGCACCCAATGTTGTT
Medtr7g407170 (847) TTTAGATATGAAAAATATTCAAATGAAAATGGGAAACATGCACCCAATGGTGTT
Medtr3g011590 (856) TTTGGAAATTCAAAATATTCAAATGAAAATGGGAAACACACATCTAATGGTGTT
Medtr3g019820 (826) TTCAGAAATGCAAAATATTCAAATGAAAATGGGAAACACACACTGAATGGTGTT
Medtr3g019900 (862) TTCAGAAATGCAAAATATTCAAATGGAAATGGGAAACACACAACGAATGGTGTT
Medtr3g019930 (862) TTCAGAAATGCAAAATATTCAAATGGAAATGGGAAACACACAACGAATGGTGTT
Medtr3g019980 (856) TTCAGAAAAGCCAAACATTCAAATGGAAATGGGAAACACACACCGAATGGTGTT
Medtr3g020100 (853) TTCAGAAATGTAAAATTTTCAAATGGTAATGGGAAACACACACCAAATGGTGTT
Medtr3g020740 (856) TTTAAAAATTAA------------------------------------------
Medtr3g020760 (856) TTTAAAAATGAAAAATATTCAAATGAAATTGGGAAACACACACCTAATGGTGTT
Medtr6g045287 (859) TTTGAATATGAAAAATATTCAAATGAAAATGGGAAACATGCACTCAATGGTGTT
Medtr7g024250 (859) TTTGGATATGAAAAATATTCAAATGAAAATGGGAAACATGCACCCAATGATGTT
    Consensus (865) TTTAGAAATGAAAAATATTCAAATGAAAATGGGAAACACACACC AATGGTGTT
                                                                         Section 18
            (919) 919        930            945
Medtr2g075100 (913) TGA------------------------
Medtr7g028880 (913) TGA------------------------
Medtr7g407170 (901) TGA------------------------
Medtr3g011590 (910) TGA------------------------
Medtr3g019820 (880) TGA------------------------
Medtr3g019900 (916) TGA------------------------
Medtr3g019930 (916) TGA------------------------
Medtr3g019980 (910) TGA------------------------
Medtr3g020100 (907) TGA------------------------
Medtr3g020740 (868) ---------------------------
Medtr3g020760 (910) TGA------------------------
Medtr6g045287 (913) TCAAACATTCGGGCAAAAATTATATAA
Medtr7g024250 (913) TGA------------------------
    Consensus (919) TGA
```

Figure S2. The stages of nodule development in *M. truncatula*. The stage of 5 dpi is characterized by cell division in the middle and inner cortex, at 7-9 dpi the nodule primordia emerge from the root. At 12 dpi vascular bundles and the meristem is being formed. The formed vascular bundles and nodule meristem are observed at 15 dpi. Infected cells are distinguished at 15-18 dpi, the pink mature nodule can be observed at 21 dpi. Bars, 100 µm. Thickness of sections, 50 µm.

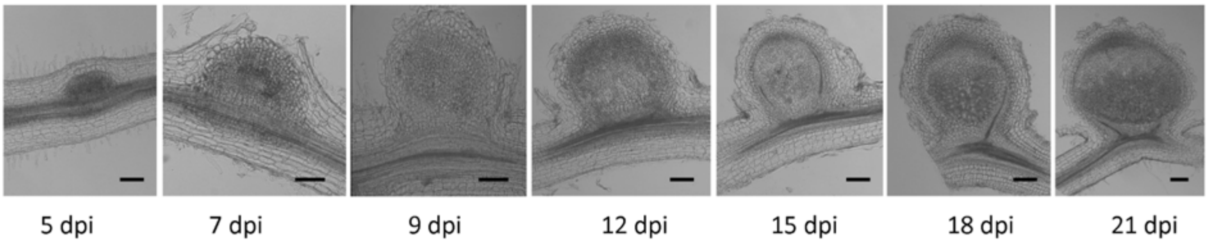

Figure S3. Expression of *MtIPT* genes in different nodule zones according to LCM-RNA-seq data, publicly available on the INRA website (https://iant.toulouse.inra.fr/symbimics/). Meristematic zone (FI), distal and proximal infection zone (FIId and FIIp), inter-zone (IZ) and fixation zone (ZIII).

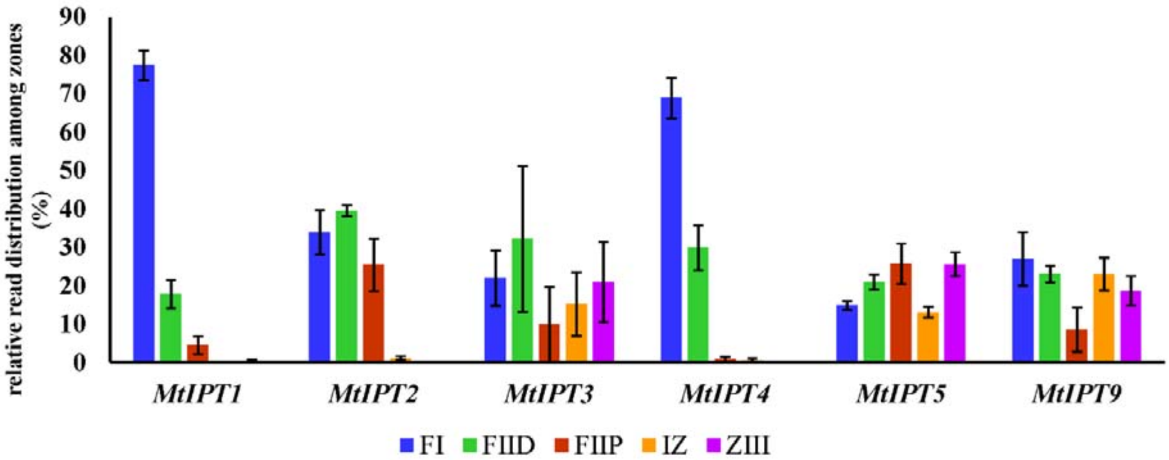

Figure S4. Relative expression of *MtIPT* genes in second leaves at different days post inoculation (3, 5, 7, 10 dpi). A and B are results of two different biological repeats. Results are mean ± SEM of three technical repeats in one biological repeat.

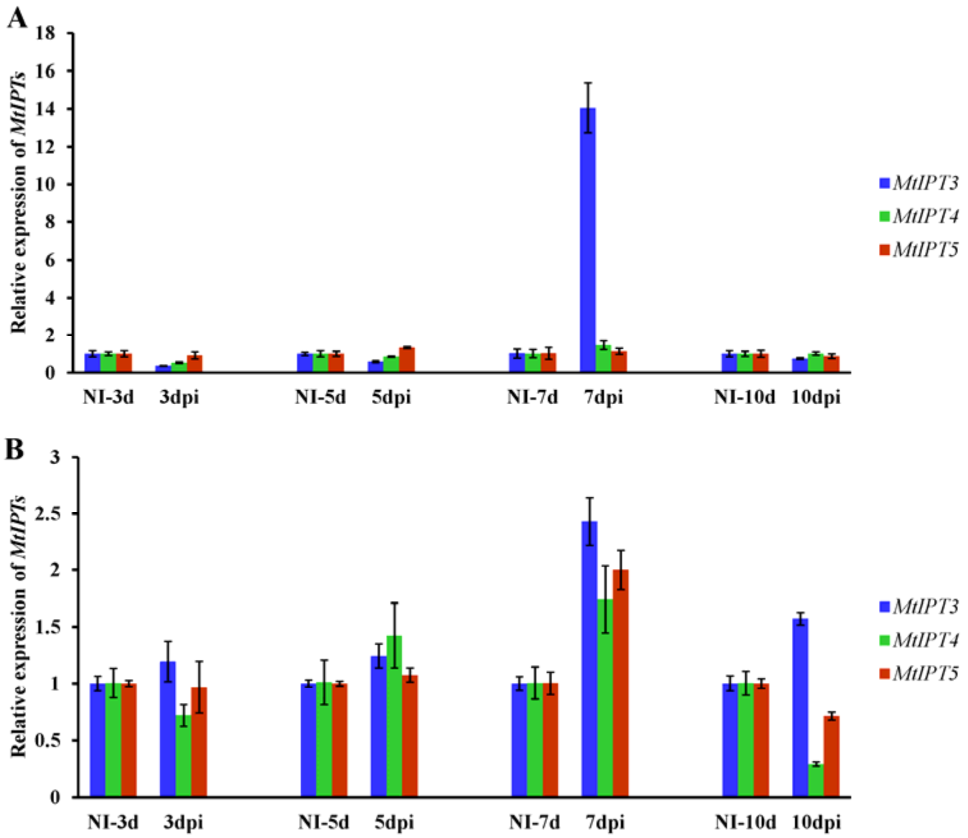

Figure S5. Relative expression of *MtIPT* genes in first leaf at different days post inoculation (3, 5, 7, 10 dpi). Results are mean ± SEM of three technical repeats in one biological repeat out of three independent experiments.

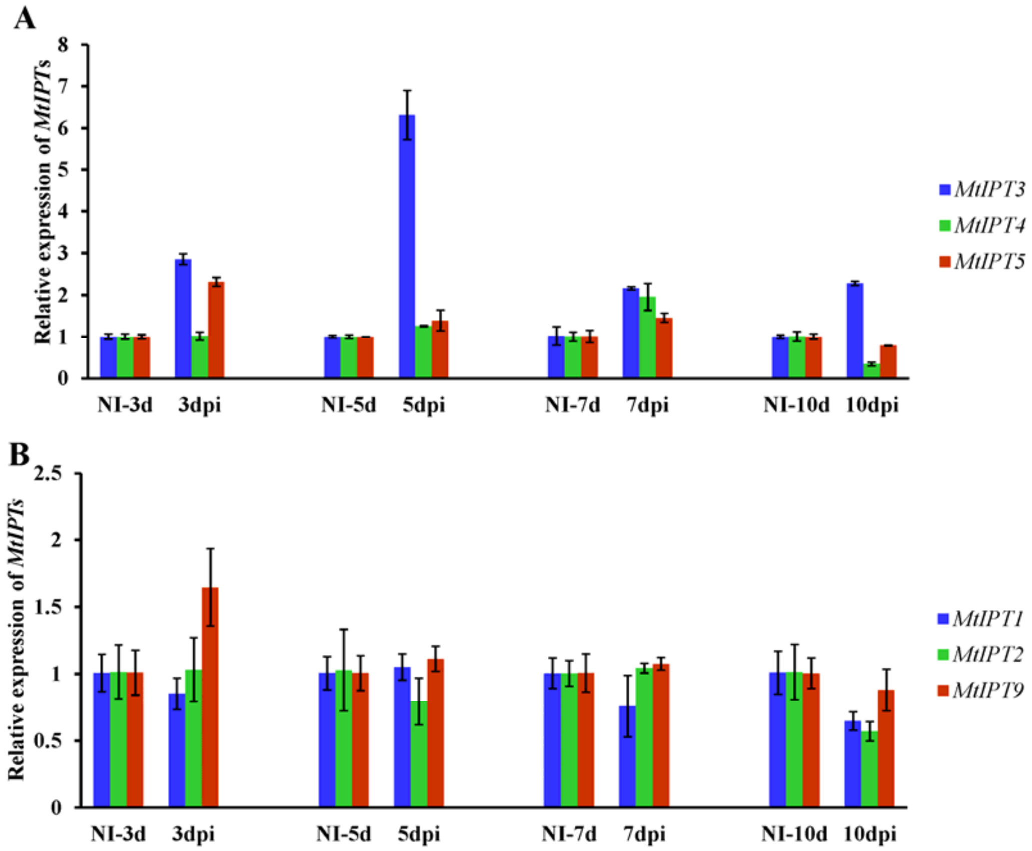

Figure S6. Relative expression of *MtIPT3*, *MtIPT4* and *MtIPT5* in first leaf of *sunn-3* mutants at different days post inoculation (3, 5, 7, 10 dpi) in comparison with non-inoculated plants (NI). Results are mean±SEM of three technical repeat of one biological repeat representative for three independent experiments.

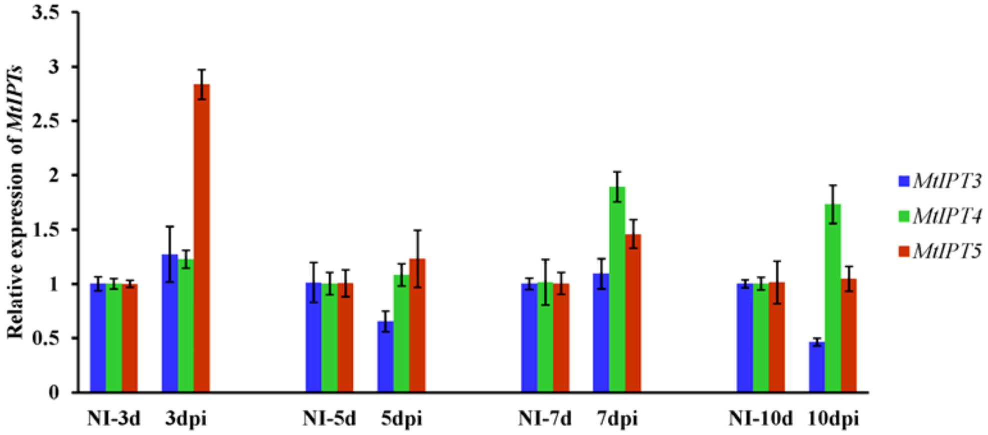

Figure S7. Relative expression of *MtIPT3*, *MtIPT4* and *MtIPT5* in second leaves of *sunn-3* mutants at different days post inoculation (3, 5, 7, 10 dpi) in comparison with non-inoculated plants (NI). Results are mean±SEM of three technical repeat of one biological repeat representative for three independent experiments.

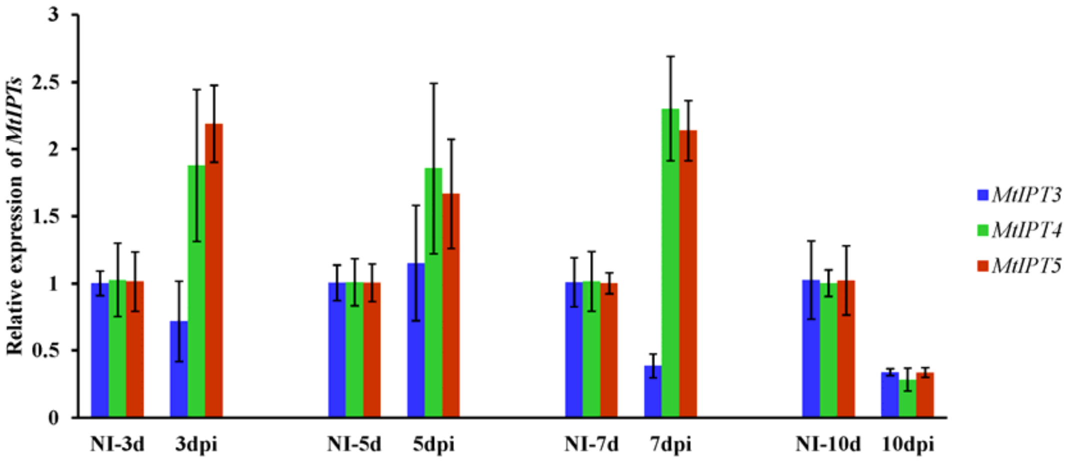

Figure S8. qRT-PCR expression analysis of *MtIPTs* in uninoculated roots (NI) and at different days post inoculation (dpi) in *sunn-3* mutants. The relative expression was normalized against constitutively expressed *Medicago truncatula* actin gene. Results are mean±SEM of three technical repeat of one biological repeat, representative for three independent experiments.

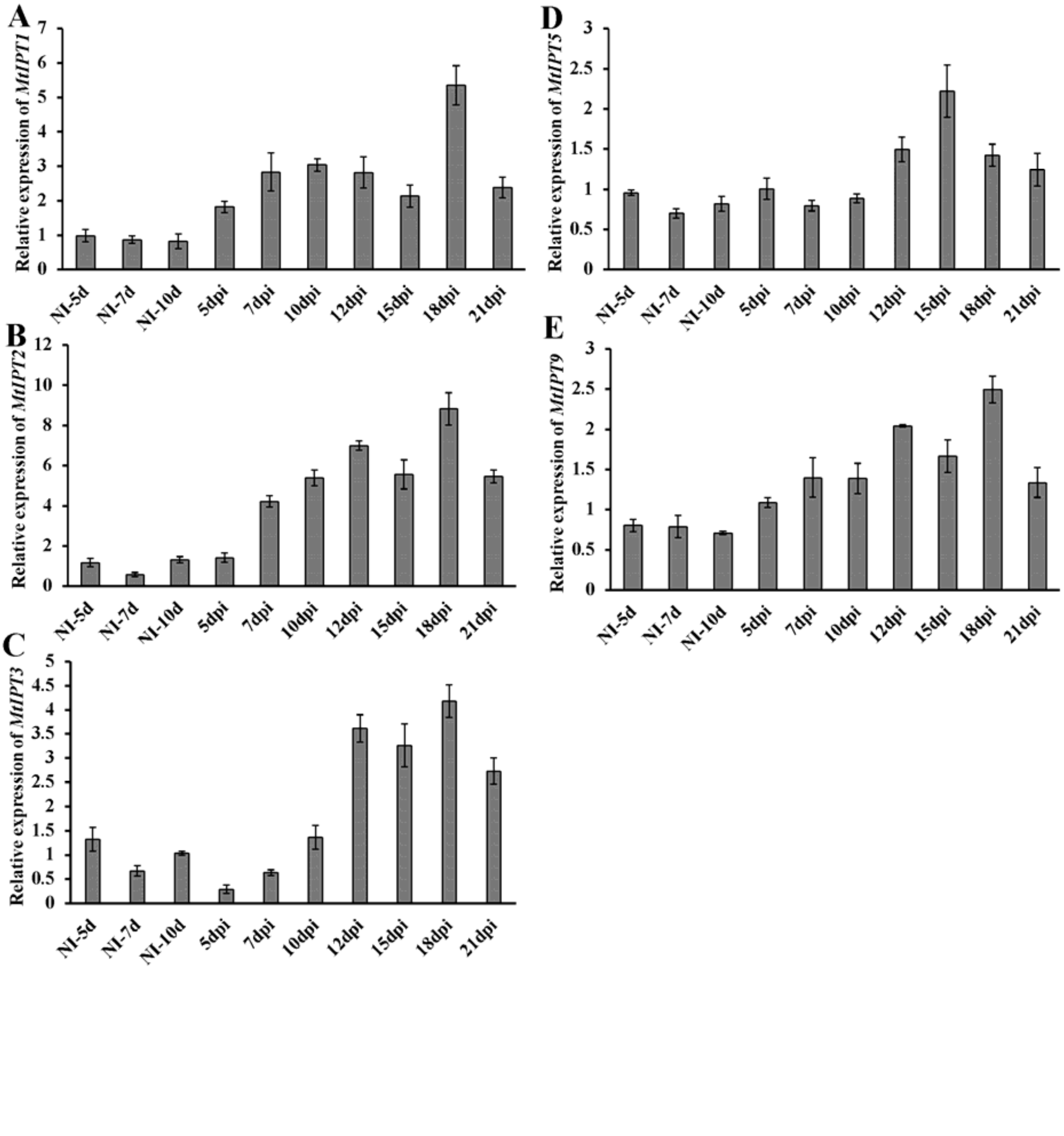

Table S1. Primer used for RT-PCR

1	*MtIPT1*	Medtr1g110590	5’-TCGCCACCCTCTTCCCTTAC-3’	5’-CCGACAACACAGGAAACGAAA-3’
2	*MtIPT2*	Medtr4g117330	5’-GTGTCGGGTGCTGTTGCTT-3’	5’-GTAGCCATCGTCGCCTGTTC-3’
3	*MtIPT3*	Medtr1g072540	5’-TCCTCCAAATCACCAAACATAAAA-3’	5’-CGCCCATCACCAACACTACA-3’
4	*MtIPT4*	Medtr2g022140	5’-GCCACCGAAACGCACCTTA-3’	5’-ATTCCCTGTCCCACCACCTC-3’
5	*MtIPT5*	Medtr4g055110	5’-AGATGACTCGACGGATGATTTGA-3’	5’-AACTTTGCTGGGAAGAACACCA-3’
6	*MtIPT9*	Medtr2g078120	5’-CAGTTGGTGGTAAAAGCAGGTGA-3’	5’-CCGAAAAGCAGAAGGAGGTGA-3’
7		Medtr2g075100	5’-GAGTGGATGAAATGGTTGAGGGT-3’	5’-GCATCATCAATGCCACTTTTCTT-3’
8		Medtr7g028880	5’-GAAGGGCTATTGGGGTTCCT-3’	5’-CCTTGCTTGGCTTAATCACGC-3’
9		Medtr7g407170	5’-CGCATCACATATTAGGCATCATCA-3’	5’-TGAAATGGCTGGGTCCTCAA-3’
10		Medtr7g024250	5’-TGTTCGATTCCGCATCACTT-3’	5’-CCTGCCTCTACCATTTCATCG-3’
11		Medtr6g045287	5’-TGGTTGAGTCAGGGATGGTTG-3’	5’-TTTGGTTTTTCTGATAGCTTCGGC-3’
12		Medtr3g011590	5’-GACGTGTCTTTGCCTATTCTATG-3’	5’-TCGGATCTTCGAGAGTTGATTT-3’
13		Medtr3g019820	5’-TGTCACCAATAAGGTGCAGGA-3’	5’-GAGCCAAATAAAACAGCAATCG-3’
14		Medtr3g019900	5’-TCAATTCCACATCATATACTAGGCATC-3’	5’-CTGCCGCAACCATTTCAC-3’
15		Medtr3g019980	5’-TACAACGGGAACTGGGAAGA-3’	5’-CATTTTGAGTTATGAGGTCTAAAGCC-3’
16		Medtr3g020100	5’-GAGGACCCAACTATTGCATTTCG-3’	5’-TCCGCACCAGGTACAAAGAA-3’
17		Medtr3g020740	5’-CGTTGTTCAATTCCTCATCATC-3’	5’-CCCCAACCATCTCATCAACTC-3’
18		Medtr3g020760	5’-AAAAATTAGTTGACGACCCAGT-3’	5’-CTTTTGTGTTGTCTGCTCCA-3’
19		Medtr7g024230	5’-TTCAATTCCACATCCATTTTATTCAA-3’	5’-ACTCAAGCTCTGCCTCAACCA-3’
